# ThermoEnergy Ammonia Recovery Process for Municipal and Agricultural Wastes

**DOI:** 10.1100/tsw.2001.287

**Published:** 2001-10-23

**Authors:** Alex G. Fassbender

**Affiliations:** ThermoEnergy Corporation, Richland, WA 99352, USA

**Keywords:** agricultural waste, Ammonia Recovery Process, ARP, ammonia, ammonium sulfate, anaerobe, anaerobic, animal feeding, biogas, biogasification, concentrated animal feeding operation, CAFO, chemisorption, dairy waste, fermentation, methanogen, methane, methanogenesis, municipal sludge, rendering, zinc

## Abstract

The Ammonia Recovery Process (ARP) is an award-winning, low-cost, environmentally responsible method of recovering nitrogen, in the form of ammonia, from various dilute waste streams and converting it into concentrated ammonium sulfate. The ThermoEnergy Biogas System utilizes the new chemisorption-based ARP to recover ammonia from anaerobically digested wastes. The process provides for optimal biogas production and significantly reduced nitrogen levels in the treated water discharge. Process flows for the ammonia recovery and ThermoEnergy biogas processes are presented and discussed. A comparison with other techniques such as biological nitrogen removal is made. The ARP technology uses reversible chemisorption and double salt crystal precipitation to recover and concentrate the ammonia. The ARP technology was successfully proven in a recent large-scale field demonstration at New York City’s Oakwood Beach Wastewater Treatment Plant, located on Staten Island. This project was a joint effort with Foster Wheeler Environmental Corporation, the Civil Engineering Research Foundation, and New York City Department of Environmental Protection. Independent validated plant data show that ARP consistently recovers up to 99.9% of the ammonia from the city’s centrate waste stream (derived from dewatering of sewage sludge), as ammonium sulfate. ARP technology can reduce the nitrogen (ammonia) discharged daily into local bodies of water by municipalities, concentrated animal farming operations, and industry. Recent advances to ARP enhance its performance and economic competitiveness in comparison to stripping or ammonia destruction technologies.

## INTRODUCTION

A significant factor contributing to the imbalance of nitrogen in the ecosystem is the loss of nitrogen compounds to the environment from current treatment and disposition of human and animal wastes and fertilizer runoff. Just 70 years ago, Fritz Haber and Karl Bosch shared the 1931 Nobel Prize for their development of industrial-scale ammonia production. Many scientists and historians consider this technological development, which enabled the "green revolution" in agriculture, to be the most important of the 20$^{th}$ century. The discovery of ammonia production and the subsequent agricultural revolution enabled the civilization we now have.

When excess ammonia finds it way into waterways it creates conditions that are unsafe for human health and the environment. Through recent improvements in analytical measuring methods, we now know that the effects of ammonia at even dilute concentrations are alarming. After entering waterways, it fuels algae growth and causes oxygen-depleting conditions such as hypoxia and eutrophication. In groundwater, ammonia oxidizes to nitrate and contaminates wells and drinking water. From dead fish, to toxic algae blooms, to blue babies, ammonia from sewage treatment, livestock feeding operations, and rendering plants affects large cities and rural communities alike. In addition to sewage treatment releases, the magnitude of contamination by excess livestock manure is beginning to unfold. Animal feeding operations in the U.S. produce a staggering 134 times as much manure waste as do humans. Past measuring and monitoring techniques have underestimated the amounts of nitrogen being discharged into the air and into watersheds, and the amount of land and vegetation needed to utilize the nitrogen in a continuous and sustainable manner. Excess animal waste spread on fields leaches ammonia into the vadose zone on its way to the groundwater. There, it oxidizes to nitrate where, for example, 10 parts per million (ppm) of ammonia becomes 36.5 ppm of nitrate. High nitrate levels in drinking water lead to blue baby syndrome, or methemoglobinemia, a potentially fatal condition.

Nitrogen-containing waste from municipal and agricultural operations is typically wet, odorous, loaded with microorganisms, and costly to handle, store, and transport. Livestock wastes are typically many times more concentrated than municipal sewage, with ammonia concentrations of 10,000 ppm or more. At these elevated ammonia concentration levels, the normal biological breakdown of animal waste into biogas is greatly inhibited by ammonia's toxic effects. By removing ammonia as the manure is digested, ThermoEnergy's Ammonia Recovery Process (ARP) technology allows natural microbiological processes to break down animal wastes more efficiently. This is true for both aerobic and anaerobic systems. Sewage and animal-based wastes are produced on a continuous basis, whereas crops in temperate zones can use nitrogen fertilizer for only a portion of the year. There is a structural and fundamental timing dislocation between the production of fixed nitrogen from biological wastes and the ability of crops to utilize the fixed nitrogen.

A 1996 survey[1] showed that 40% of the U.S. rivers and waterways surveyed are contaminated by excess nutrients like ammonia and its oxidation product, nitrate, creating conditions that are unsafe for human health and the environment. In response, strategies for reversing pollution, such as the 1998 Clean Water Initiative, propose aggressive prevention efforts and restoration progress. ThermoEnergy's ARP solves the ammonia problem by efficiently removing and recovering it from the waste rather than discharging it or vaporizing it into the atmosphere. The ARP technology fulfills the urgent need to improve water quality. It does not require large amounts of energy or an array of chemicals; it uses little space; it has no fouling or odor problems; and it is integrated to enhance the performance of existing waste treatment facilities. A happy consequence of the recovery of ammonia from human- and animal-generated wastes is enhanced production of energy in the form of biogas. Ammonia recovery from industrial livestock operations will enable the agriculture industry to expand production, meet environmental regulations, maintain a clean environment, recycle the main chemical of the green revolution, and bring the human impact on the planetary nitrogen cycle under sustainable management.

## PROCESS DEVELOPMENTS

The patent-pending ARP recovers ammonia from aqueous streams, such as the liquid product resulting from dewatering or centrifuging anaerobically digested animal waste and sewage sludge. This stream, known as the *centrate*, contains approximately 600 to 3000 ppm dilute ammonia. ARP is the first cost-effective technology to recover ammonia from water at low concentrations (i.e., diluted ammonia). The technology evolved from laboratory-scale ammonia recovery research by Battelle Memorial Institute and was licensed to ThermoEnergy Corporation. The features of the ARP technology prompted Foster Wheeler Environmental Corporation, an international environmental consulting, engineering, and construction firm, to enter into a joint venture with ThermoEnergy Corporation to demonstrate and market the process. The City of New York provided a site at the Oakwood Beach Water Pollution Control Plant on Staten Island and detailed an engineer to work with the demonstration team. During the demonstration, the ARP consistently maintained 90% removal efficiency during extreme operating conditions and weather fluctuations. The process was further proven in tests by the New York City Department of Environmental Protection (NYCDEP) and by the Environmental Technology Evaluation Center (EvTEC, a joint effort of the U.S. Environmental Protection Agency and the Civil Engineering Research Foundation) using blind, side-by-side comparison samples, which showed no significant statistical variations in the 90% ammonia recovery. Fig. 1 shows a process flow diagram of the New York demonstration system. The recovered ammonia was converted it to a storable and marketable commodity: standard, dry ammonium sulfate fertilizer. A peer-reviewed third-party independent evaluation was conducted by EvTEC.[2]

To begin the ARP, a proprietary resin loaded with zinc selectively chemisorbs ammonia onto ion exchange columns at a rate of 8 g of ammonia per liter of resin. The zinc-loaded ARP ion exchange resin creates a chemisorption driving force that is extremely effective and selective in removing ammonia from aqueous streams. The columns are regenerated using a low-pH solution of sulfuric acid and zinc sulfate. The regeneration solution is used repeatedly, where the ammonia concentration builds up to 20,000 ppm. In the demonstration plant, steam was used to further concentrate this solution. The concentrated ammonia-laden regeneration solution is cooled to allow zinc ammonium sulfate hexahydrate crystals to form. These large, easily separated double salt crystals are then roasted to drive off ammonia. The resultant ammonia gas is recovered in a sulfuric acid scrubber where ammonium sulfate forms. The recovered ammonium sulfate crystals are dried and bagged, and are immediately available as a common and widely used crystalline fertilizer. The zinc sulfate crystals remaining in the roaster are recirculated and used to prepare fresh column regeneration solution. No secondary waste is produced during ammonia recovery.

A key to the success of ARP is the use of zinc sulfate in the regeneration solution. The zinc sulfate–sulfuric acid solution used in the ARP technology keeps the zinc on the column but removes the ammonia. Traditional gas-liquid stripping technologies have intrinsic mass transfer limitations that cause operating costs to increase dramatically as the ammonia concentration decreases. In contrast, the ARP uses chemisorption, a fundamentally different, liquid-phase technique that has demonstrated reduction of ammonia concentrations to undetectable levels.

By achieving a 20 to 1 initial concentration, and by using a double-salt crystallization technique to recover the ammonia in an even more concentrated state, the process minimizes the quantity of water evaporated. Consequently, the ARP removes am-

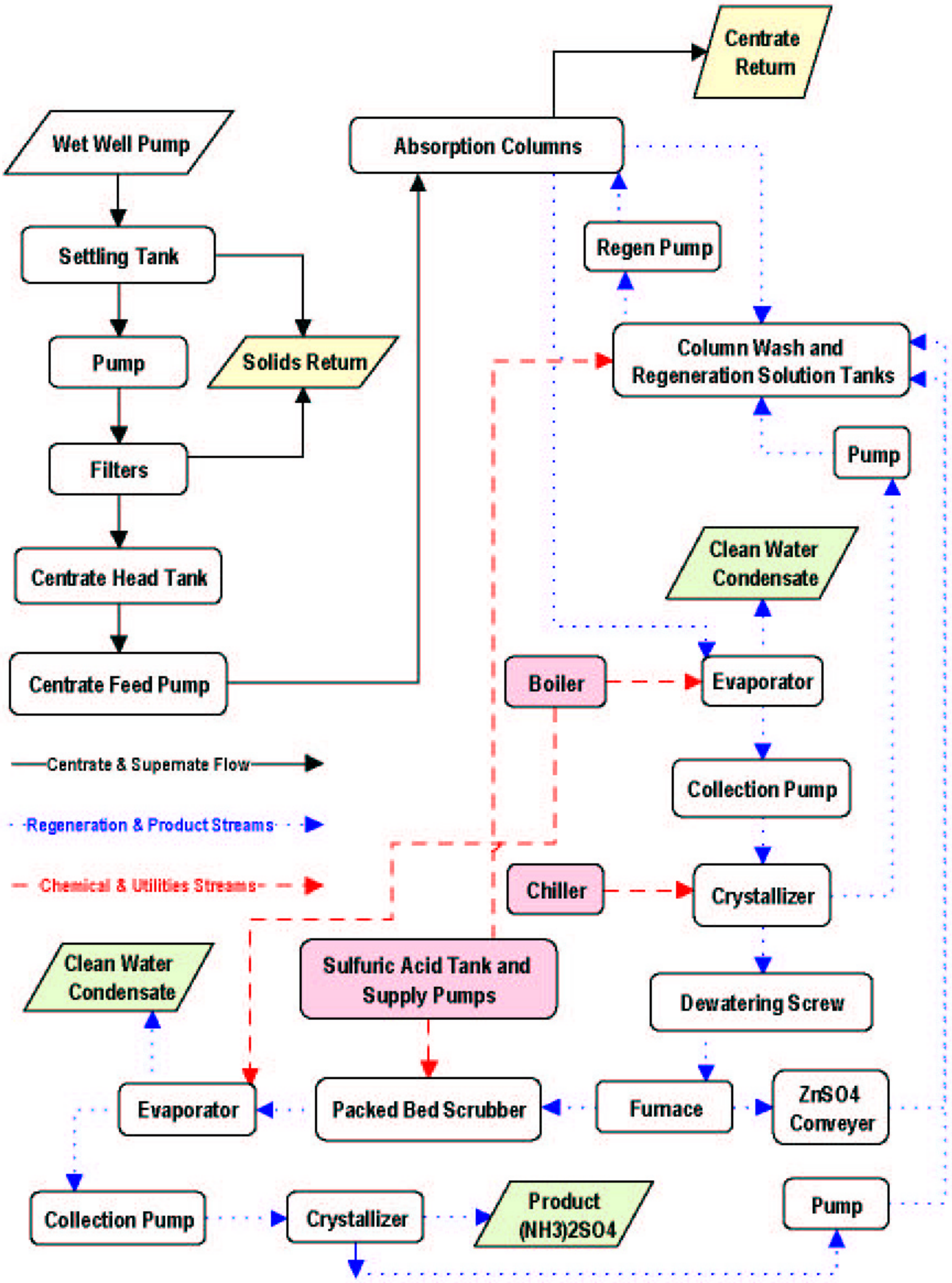

**FIGURE 1.** Process flow schematic of the New York City ARP demonstration.

monia as a concentrated product at lower costs and energy requirements than steam or water stripping. Also unlike stripping processes, the chemisorption driving force does not diminish as the concentration of ammonia decreases. Fig. 2 summarizes the process chemistry.

Over the past several decades, extensive scientific and engineering work has been conducted on the biogasification of waste materials. The fundamental technique relies on the anaerobic digestion or fermentation process. Anaerobic digestion of biomass materials proceeds in three distinct and sequential pathways: hydrolysis, acetogenesis, and methanogenesis. The anaerobic microorganisms that conduct the first two steps, the hydrolyzers and acetogens, break the complex biomass molecules down into small chain molecules. Proteins are hydrolyzed into proteoses, peptones, and polypeptides. These compounds are further broken down into ammonia and small-chain fatty acids such as acetic acid, butyric acid, propionic acid, and lactic acid. Many common bacteria contain the urease enzyme that hydrolyzes urea

| | |
|---|---|
| Centrate Solution Equilibrium: | $NH_4^+ / NH_3 + H^+$ |
| Ammonia Adsorption onto Column: | $R\text{-}Zn^{++} + 2NH_3 \quad R\text{-}Zn^{++}{:}(NH_3)_2$ |
| Ammonia Stripping from Column | $R\text{-}Zn^{++}{:}(NH_3)_2 + 2H^+ \quad R\text{-}Zn^{++} + 2NH_4^+$ |
| Zinc Adsorption Equilibrium on Column: | $R\text{-}2(H^+) + Zn^{++} / R\text{-}Zn^{++} + 2H^+$ |
| Double Salt Gets Ammonia out of Solution: | $2NH_4^+ + Zn^{++} + 2SO_4^{--} + 6H_2O /$ |
| | $(NH_4)_2SO_4.ZnSO_4\ .6H_2O$ ppt |
| Solubility of 7 gms/100 gms @ 0°C and 42 gms/100 gms @ 80°C | |
| Separating the Ammonia from the Zinc: | $(NH_4)_2SO_4.ZnSO_4\ .6H_2O$ + heat (330°C) |
| | $NH_{3(g)} + ZnSO_{4(s)} + SO_{3(g)} + 7H_2O_{(g)}$ |
| Forming Ammonium Sulfate: | $2NH_{3(g)} + H_2SO_{4(l)} \quad (NH_4)_2SO_4$ Solution |
| | $SO_{3(g)} + H_2O_{(l)} \quad H_2SO_{4(l)}$ |
| Dehydrate and Crystallize | $(NH_4)_2SO_4$ Solution $(NH_4)_2SO_4$ Crystal + $H_2O_{(g)}$ |

**FIGURE 2.** ARP chemistry sequence.

into carbon dioxide and ammonia.[3,4] The anaerobic microorganisms that perform the hydrolysis and acetogenesis functions are highly resistant to ammonia. Anaerobic fermentation of high-nitrogen wastes using these microorganisms have produced digested streams containing in excess of 10,000 ppm ammonia. However, the anaerobic microorganisms responsible for methanogenesis are inhibited by ammonia. Methanogenic anaerobic bacteria cease to function effectively at ammonia concentrations equal to or greater than approximately 1200 ppm ammonia.[1] Hansen et al.[6] concluded that "A threshold value was found for inhibition of the biogas process of 1.1 g-N/l free ammonia."

Technologies such as the upflow anaerobic sludge blanket (UASB) reactor, the extended granular sludge bed (EGSB) reactor, and the membrane bioreactor offer advantages in the anaerobic fermentation or digestion of wastewater or other feed stocks. These reactors allow for higher treatment rates using smaller vessels, thereby reducing capital costs. These reactors also provide for improved odor control. Still, problems associated with ammonia inhibition have made these reactors relatively unstable and difficult to operate when using feed stocks containing relatively high concentrations of nitrogen. To mitigate these problems, it has been proposed to control the carbon to nitrogen (C/N) ratio of the feed stock and to dilute the reactors with water in cases of sudden, large ammonia overloads. These proposals still suffer from a number of disadvantages. For example, adjusting the ammonia concentration in a reactor by adjusting the C/N ratio of the feed stock is a slow process; it can be difficult to accurately determine the C/N ratio; and adjusting the C/N ratio may prove to be insufficient to handle feed stocks that are prone to generate relatively high ammonia concentrations during anaerobic digestion. Dilution of a reactor with water also has a number of disadvantages. For example, diluting the reactor with water may seriously decrease the reactor's biogas production for extended periods of time and will typically lead to increased dewatering costs. Dilution of an existing feed stock increases the required reactor volume for digestion of that feed stock. An existing reactor would have a decreased capacity for treating a given feed stock if that feed stock were diluted.

Toward the fulfillment of these and other objects and advantages, the ThermoEnergy Biogas System uses an ammonia recovery system in combination with anaerobic digesters to maximize biogas production. In one configuration of this approach, a single anaerobic digester may be used: a portion of the effluent stream is removed from the reactor, treated to remove ammonia, and a portion of the treated water recycled to the digester at a rate designed to avoid ammonia inhibition problems within the digester. The system may be operated under mesophilic or thermophilic conditions depending on the amount of heat available.

The patent-pending ThermoEnergy Biogas System schematic is shown in Fig. 3. Raw waste and wash water is comminuted and preheated, then sent to the anaerobic digester. The digester is set up with ancillary equipment such as a membrane or sand filter so that it continually draws an ammonia-laden liquid stream from the digester. This ammonia-laden stream is processed to remove the ammonia and a portion of the low-ammonia stream recycled back into the digester. The pH of this stream is adjusted to optimize the pH in the mixed reactor feed. This recycle stream is a fairly clean water stream than can be used to recover heat from the exhaust of the combined heat and power unit. Using this relatively clean stream as a heat transfer stream has the advantage that it is easier to pump through a heat exchanger than the animal waste stream. The biogas is utilized in combined heat and power facility that can be as simple as a small internal combustion engine. Excess sludge is dewatered and air-dried. The bulk of the volatile carbon and nitrogen is captured and reused in the biogas and ammonium sulfate respectively while the phosphorus is sequestered in the relatively inert dried excess sludge.

This system is designed to:

- Recover ammonia in a concentrated, useful form
- Provide ammonia removal prior to methanogenesis to optimize the C/N ratio and avoid ammonia-inhibition problems
- Provide a system that generates energy from the enhanced biogas production
- Provide a thoroughly digested, stable biosolid material

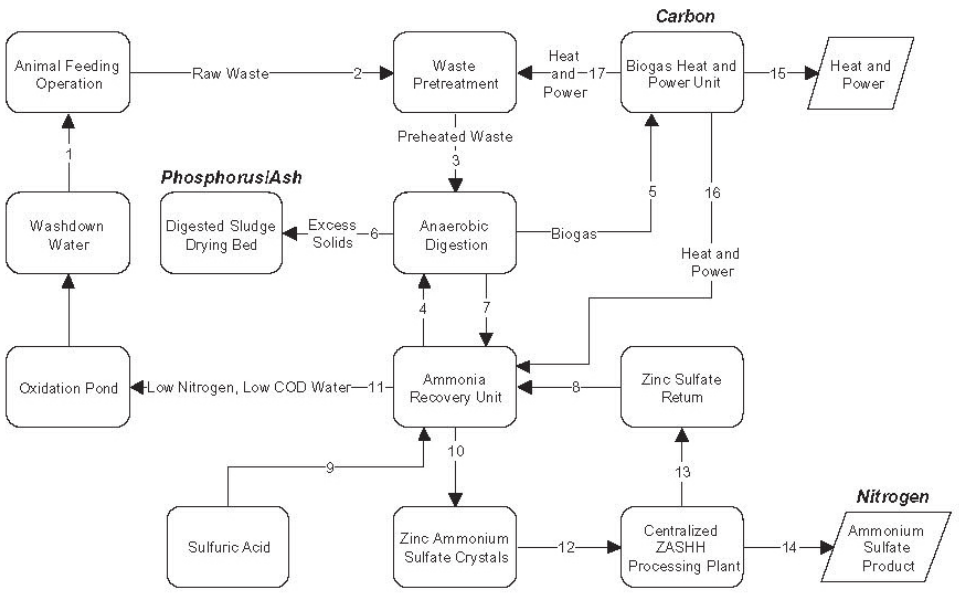

**FIGURE 3.** ThermoEnergy Biogas System process flow schematic.

## IMPLEMENTATION MODALITIES AND ECONOMICS

The ARP can be implemented in centralized and distributed modes. In the centralized mode, all of the unit operations are conducted to produce recovered ammonium sulfate from wastewater. Detailed designs have been developed for a range of ARP plants. Table 1 shows the estimated capital and operating costs for a range of centralized ARP plants.

Large animal feeding operations and municipal sludge dewatering operations lend themselves to the centralized mode of implementation. Smaller municipal treatment plants and animal feeding operations lend themselves to the implementation of the ARP in a distributed mode.

In a distributed mode, the ARP is separated into two parts: an ammonia capture portion and a regeneration portion. The equipment utilized in the ammonia capture portion consists of the equipment necessary to produce the double salt crystals, and is scalable over a wide range. ARP ammonia capture chemisorption equipment is similar to ion exchange equipment; an example of a small-scale ion exchange resin column is the home water softener. The equipment used in a regeneration portion to convert the double salt crystals into ammonium sulfate crystals and zinc sulfate crystals has significant economies of scale. The regeneration portion unit operations would be conducted at a centralized facility and receive double salt crystals from distributed ammonia capture systems.

## FUTURE WORK

The plan is to integrate anaerobic digestion of concentrated animal feeding operation (CAFO) wastes with the recovery of biogas and ammonium sulfate fertilizer. The objective of the technical development effort will be to maximize the production of biogas and the recovery of ammonia from CAFO wastes. This will be accomplished by controlling the concentration in the anaerobic reactor and configuring the reactor to maximize biomass age and mixed liquor suspended solids concentrations.

There are a number of candidates for the anaerobic digestion reactor. Traditional UASB or anaerobic baffled reactors combined with a settling and filter system represent the first and most common type of anaerobic system candidate. The second type of candidate is a membrane bioreactor. Near-term activities should

**TABLE 1**
**Centralized Ammonia Recovery Plant Budgetary Estimates**

| GPM | $[NH_3]$ ppm | No. Resin Beds | Size Resin Beds | Cap. Cost, $MM | O&M, cents/gal |
|---|---|---|---|---|---|
| 250 | 1000 | 3 | 8′ | 5.6 – 10.6 | 2.6 |
| 550 | 1000 | 3 | 12′ | 9.3 – 17.0 | 1.5 |
| 1000 | 1000 | 3 | 16′ | 15.2 – 24.3 | 1.2 |
| 2100 | 650 | 7 | 16′ | 35.8 – 44.0 | 1.0 |

consider the first type of candidate as these units are generally familiar to the industry and the phosphorus is captured either in the biosolids or by alum precipitation. Long term, membrane bioreactors may offer the advantages of minimizing the complexity of the anaerobic reactor system and the number of unit operations. This type of reactor also enables extended sludge ages and produces a clear low suspended solids wastewater suitable for treatment in an ammonia recovery unit.

| | | |
|---|---|---|
| Received: | July | 13, 2001 |
| Revised: | September | 26, 2001 |
| Accepted: | October | 9, 2001 |
| Published: | October | 23, 2001 |

## BIOSKETCH

**Alex G. Fassbender** is Executive Vice President of ThermoEnergy Corporation (TEC) with primary responsibilities for technology deployment. Mr. Fassbender also serves as Vice President of Technology for ThermoEnergy Environmental Corporation, TEC's joint venture with Foster Wheeler Environmental Corporation and Technical Director of ThermoEnergy Power Systems LLC. In these roles he coordinates the technical aspects of merging science and engineering to produce working process systems. Mr. Fassbender received his B.S in Chemical Engineering in 1976 from the University of California, Berkeley, and his MBA in Business Economics in 1980 and M.S. in Chemical Engineering in 1988 from the University of Washington. From 1976 to 1999, Mr. Fassbender served as a Technology Commercialization Manager at Battelle Pacific Northwest National Laboratory and was responsible for investments in technology evaluation, technical, legal and business planning related to technology commercialization. He has leadership experience in pilot plant design, development and operation in the fields of wastewater treatment, energy production, hazardous waste treatment, and chemical process technology and has conducted technology applications research and techno-economic analysis. He has managed, designed, built and operated both pilot plants and bench-scale chemical process systems. Research and Development magazine presented Mr. Fassbender four R&D 100 Awards over his career. In 1988 and again in 1995, Mr. Fassbender was presented with national awards for Excellence in Technology Transfer by the Federal Laboratory Consortium.
